# DsHsp90 Is Involved in the Early Response of *Dunaliella salina* to Environmental Stress^†^

**DOI:** 10.3390/ijms13077963

**Published:** 2012-06-27

**Authors:** Si-Jia Wang, Ming-Jie Wu, Xiang-Jun Chen, Yan Jiang, Yong-Bin Yan

**Affiliations:** 1 Key Laboratory of Bio-Resources and Eco-Environment of MOE, College of Life Science, Sichuan University, Chengdu 610064, China; 2 State Key Laboratory of Biomembrane and Membrane Biotechnology, Department of Biological Sciences and Biotechnology, Tsinghua University, Beijing 100084, China

**Keywords:** *Dunaliella salina*, gene structure, haloadaption, heat shock, Hsp90, osmotic stress, structural feature

## Abstract

Heat shock protein 90 (Hsp90) is a molecular chaperone highly conserved across the species from prokaryotes to eukaryotes. Hsp90 is essential for cell viability under all growth conditions and is proposed to act as a hub of the signaling network and protein homeostasis of the eukaryotic cells. By interacting with various client proteins, Hsp90 is involved in diverse physiological processes such as signal transduction, cell mobility, heat shock response and osmotic stress response. In this research, we cloned the *dshsp90* gene encoding a polypeptide composed of 696 amino acids from the halotolerant unicellular green algae *Dunaliella salina*. Sequence alignment indicated that DsHsp90 belonged to the cytosolic Hsp90A family. Further biophysical and biochemical studies of the recombinant protein revealed that DsHsp90 possessed ATPase activity and existed as a dimer with similar percentages of secondary structures to those well-studied Hsp90As. Analysis of the nucleotide sequence of the cloned genomic DNA fragment indicated that *dshsp90* contained 21 exons interrupted by 20 introns, which is much more complicated than the other plant *hsp90* genes. The promoter region of *dshsp90* contained putative *cis*-acting stress responsive elements and binding sites of transcriptional factors that respond to heat shock and salt stress. Further experimental research confirmed that *dshsp90* was upregulated quickly by heat and salt shock in the *D. salina* cells. These findings suggested that *dshsp90* might serve as a component of the early response system of the *D. salina* cells against environmental stresses.

## 1. Introduction

The unicellular green algae *Dunaliella* is the most halotolerant eukaryote known, and can survive in media of a wide range of chemical compositions and salt concentrations ranging from 0.05 M to saturation. The genus *Dunaliella*, a member of the Polyblepharidaceae family, was first discovered by Michel Felix Dunal in 1838 and named after him in 1905 [1]. During the past over 100 years, several well-known *Dunaliella* species, including *D. salina*, have been used as model organisms to investigate the molecular mechanism of saline adaption [1,2]. The morphology of *Dunaliella* is similar to another model organism *Chlamydomonas reinhardtii* except that the former one contains no rigid polysaccharide cell wall to facilitate the rapid alternation of cell volume upon osmotic pressure. In addition to the exceptional halotolerance, some *Dunaliella* species also accumulate high concentrations of carotenoids and glycerol, which makes them widely used as the cell factory for the commercial production of *β*-carotene and glycerol [3,4]. The accumulation of carotenoids may be important for *Dunaliella* to fight against excess blue light or UV irradiation [5], while glycerol is rapidly converted from starch to maintain the homeostasis when *Dunaliella* cells are under high-salt stress [6].

The morphological and metabolic changes of *Dunaliella* cells during osmotic stress have been well-established [2]. In brief, when subject to hyperosmotic or hypoosmotic shock, an alteration occurs in the *Dunaliella* cell shape, volume and intracellular ion strength within minutes, followed by a balance of the osmolarity via adjustment of intracellular glycerol concentration achieved within 2–3 hours, and finally the cells adapt to the new situation by synthesizing salt-induced proteins. Undoubtedly, the regulation of intracellular glycerol concentration is the most important mechanism of *Dunaliella* halotolerance [6,7], and the key enzymes involved in glycerol synthesis including glycerol 3-phosphate dehydrogenase1 (GPD1) have been cloned and studied [2,8]. However, the high-osmolarity glycerol (HOG) pathway, which is well-characterized in the hyperosmotic response of *Saccharomyces cerevisiae* [9], has not been identified in *Dunaliella*. Moreover, the mechanism of *Dunaliella* halotolerance is much more complex since the cells have to adjust their metabolism state to adapt to the new environment. Recently, several proteomic or genomic studies have revealed that many genes are regulated when *Dunaliella* cells are subjected to salinity stress [10–13]. Among them, the proteins involved in protein turnover are found to be up-regulated [11]. This discovery is not surprising since the conversion of the *Dunaliella* cells’ life style requires the synthesis of new proteins, the degradation of unnecessary proteins and the stabilization of proteins against stresses by protein quality control systems. Particularly, two heat shock proteins (Hsps) with high homology to Hsp70 and Hsp90 have been identified by proteomic analysis [11].

Hsp90, a molecular chaperone highly conserved across the species from prokaryotes to eukaryotes, is abundantly expressed in higher eukaryotes and is essential for cell viability under all growth conditions. Hsp90 exists as a dimer and each subunit contains three domains: the N-terminal ATPase domain (ND) and the C-terminal dimerization domain (CD) connected by the M-domain (MD) [14]. Hsp90 is proposed to be a hub of the signaling network and protein homeostasis of eukaryotic cells [15–17]. Compared with the other general Hsps, Hsp90 specifically interact with a subset of proteins, and more than 200 substrate (also called client) proteins have been identified. These client proteins are involved in diverse physiological/pathological processes such as signal transduction, cell mobility, tumorigenesis, steroid signaling, innate immunity, heat shock response and telomere maintenance [15–18]. The client proteins, Hsp90, Hsp70 and the regulatory proteins assemble into a large multichaperone machine to regulate the client protein function and turnover [14,19]. In *Saccharomyces cerevisiae*, Hsp90 is found to be required for the high osmotic stress response and regulates a novel signaling pathway via the co-chaperone Cdc37p parallel to the HOG pathway [20,21]. Considering that Hsp70- and Hsp90-like proteins were upregulated in *Dunaliella* under halophilic conditions [11] and the importance of Hsp90 in the stress response of eukaryotic cells, it is possible that *Dunaliella* Hsp90 (DsHsp90) is involved in the halotolerance of *Dunaliella*. In this research, we cloned the *dshsp90* gene, monitored its salt-induced expression and characterized the biochemical and biophysical properties of the recombinant DsHsp90. The results herein suggested that DsHsp90 belongs to the Hsp90A family and is involved in the adaption of *Dunaliella* against extreme environmental conditions.

## 2. Results and Discussion

### 2.1. Cloning and Gene Structure of *Dshsp90* from *D. Salina*

**Figure 1:**
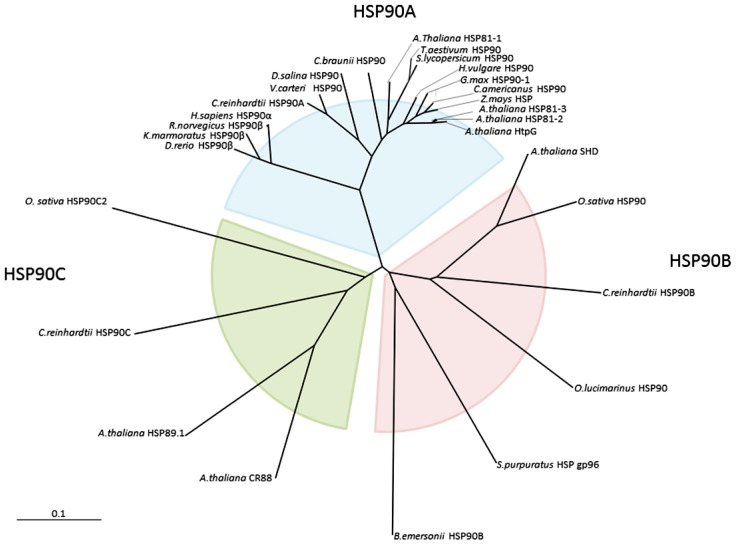
Phylogenetic tree analysis of Hsp90s. The sequences used for analysis are: *Arabidopsis thaliana* HSP81-1 (NP_200076.1), *Arabidopsis thaliana* HSP81-2 (NP_200414.1), *Arabidopsis thaliana* HSP81-3 (NP_200412.1), *Arabidopsis thaliana* HtpG (NP_200411.1), *Arabidopsis thaliana* endoplasmin-like protein (SHD) (NP_194150.1), *Arabidopsis thaliana* Chaperone protein htpG family protein (CR88) (NP_178487.1), *Arabidopsis thaliana* HSP89.1 (NP_187434.2), *Cenchrus americanus* HSP90 (ADP89126.1), *Chara braunii* HSP90 (BAH97107.1), *Chlamydomonas reinhardtii* HSP90A (XP_001695264.1), *Chlamydomonas reinhardtii* HSP90B (EDP06860.1), *Chlamydomonas reinhardtii* HSP90C (AAU10511.1), *Dunaliella salina* HSP90 (AFK31312.1), *Glycine max* HSP90-1 (NP_001236612.1), *Hordeum vulgare* HSP90 (AAP87284.1), *Oryza sativa* HSP90 (BAA90487.1), *Solanum lycopersicum* HSP90 (AAD30456.1), *Strongylocentrotus purpuratus* HSP gp96 (NP_999808.1), *Triticum aestivum* HSP90 (AEK01109.1), *Volvox carteri f. nagariensis* HSP90 (XP_002947115.1), *Zea mays* HSP (NP_001170480.1), *Blastocladiella emersonii* HSP90B (ABU45371.1), *Danio rerio* HSP90β (NP_571385.2), *Ostreococcus lucimarinus* HSP90 (ABP00103.1), *Rattus norvegicus* HSP90β (NP_001004082.3), *Homo sapiens* HSP90 α (AAH68474.1), *Kryptolebias marmoratus* HSP90β (AEM65181.1), *Oryza sativa* HSP90C2 (XP_483065.1).

The construction of the cDNA library and the generation of the ESTs were as described previously [22], and a 566 bp fragment was identified to be homologous to *Chlamydomonas reinhardtii* Hsp90A (CrHsp90A). The full length gene was obtained by 5′ and 3′ RACEs to amplify the flanking regions using the primers listed in Table S1, which resulted in a 2395 bp cDNA including an ATG start codon, a TAA stop codon, a 41 bp 5′ untranslated region (UTR) and a 263 bp 3′ UTR. The cDNA open reading frame encoded a protein composed of 696 amino acids (Accession No. JQ735968) with a predicted pI of 4.96 and an estimated molecular weight of 79619.2 Da. Hsp90 is a family of proteins with the size ranging from 80 to 110 kDa, and can be divided into five families: cytoplasmic Hsp90 (Hsp90A), endoplasmic reticulum Hsp90 (Hsp90B), chloroplast Hsp90 (Hsp90C), mitochondria Hsp90 (TRAP) and bacteria Hsp90 (HtpG) [17]. The phylogenetic tree (Figure 1) constructed according to the sequences of various Hsp90s clearly indicated that DsHsp90 belongs to the cytoplasmic Hsp90, and has a close evolutionary relationship to the Hsp90As from algae.

**Figure 2:**
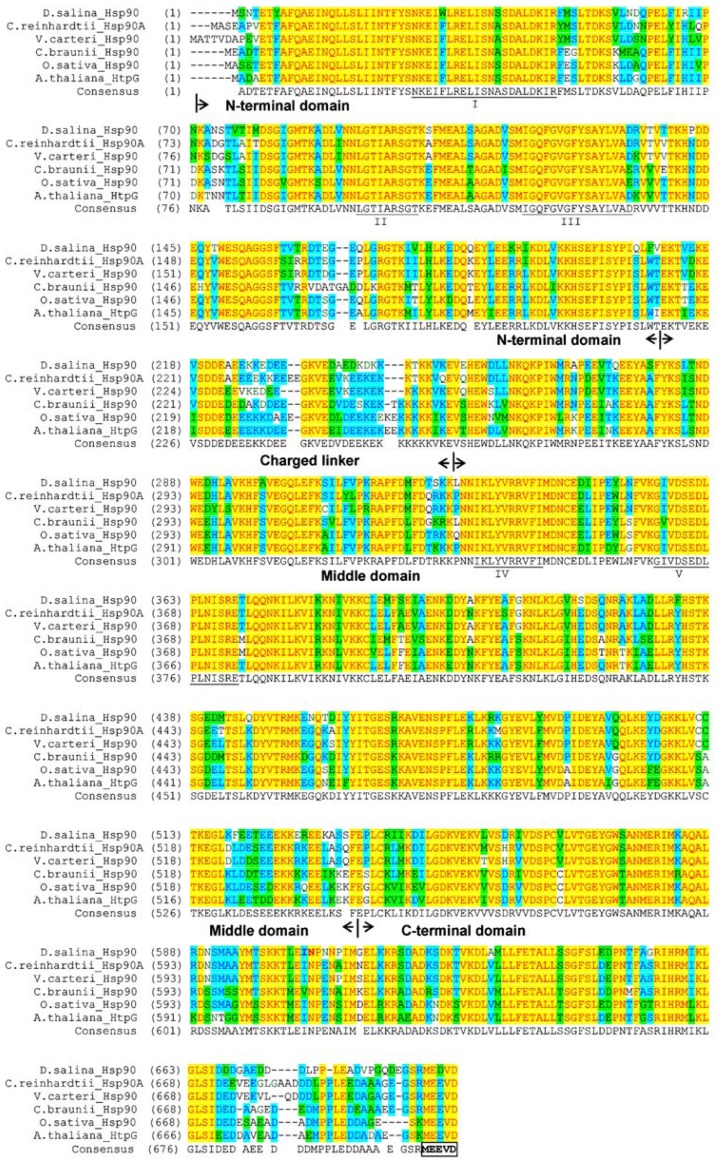
Sequence alignment of Hsp90s. The sequences used for analysis are from *D. salina* Hsp90 (JQ735968), *C. reinhardtii* Hsp90A (XP_001695264.1), *V. carteri* Hsp90 (XP_002947115.1), *C. braunii* Hsp90 (BAK08741.1), *O. sativa* Hsp90 (NP_001063500.1), *A. thaliana* HtpG (NP_200411.1). Five signature sequences (I–V) characteristic of the cytosolic Hsp90A family according to Gupta [23] are highlighted by underlining. The C-terminal MEEVD sequence, which is also a characteristic motif of Hsp90A members, is highlighted by a box.

Sequence alignment indicated that DsHsp90 shared the highest homology with CrHsp90A (83.3%) and Hsp90 from *Volvox carteri f. nagariensis* (81.8%). The plant Hsp90As are highly conserved and the similarity of DsHsp90 to the other plant Hsp90s was more than 75%. As shown in Figure 2, the three structural/functional domains (ND, MD and CD) as well as the charged linker region were well-conserved in DsHsp90. Consistent with the phylogenetic tree analysis in Figure 1, DsHsp90 belonging to the cytosolic Hsp90 family was also indicated by the absence of any predictable organelle-specific N-terminal transit peptide sequence. Moreover, the five conserved signature sequences of cytosolic Hsp90As were well-conserved in DsHsp90. Motifs II-V were almost identical to those identified previously [23], while the Phe conserved in the other plant Hsp90As (NKEIFLRELISNASDALDKIR) was replaced by another aromatic residue Trp in motif I of DsHsp90 (NKEIWLRELISNASDALDKIR). Consistent with previous sequence analysis of Hsp90As, the charged linker and the C-terminus were the most variable regions in plant Hsp90s, which provided Hsp90 specificity in binding client proteins. Notably, the highly conserved motif MEEVD at the C-terminus characteristic of the Hsp90A family, which is crucial to the binding of co-chaperones containing tetratricopeptide repeats to cytosolic Hsp90s [17], was replaced by MEDVD in DsHsp90. Nonetheless, the similar properties of the acidic amino acid residues Asp and Glu implied that the motif MEDVD in DsHsp90 also maintained the conserved function of the C-terminus in Hsp90As.

**Figure 3:**
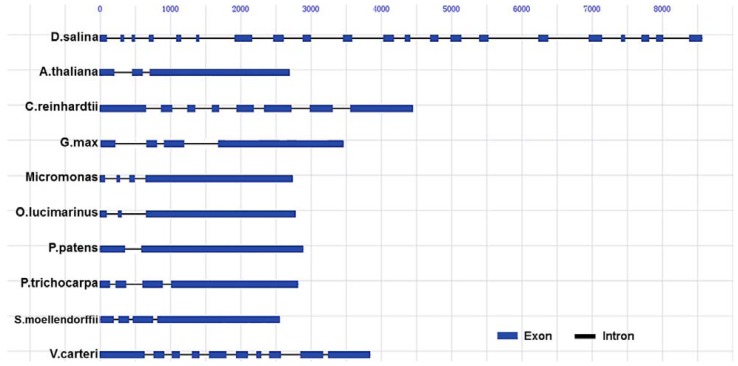
Distribution of exons and introns in the genomic DNA of *hsp90s.* The sequences used for analysis are from *D. salina* (JQ735969), *A. thaliana* (NM_124982.2), *C. reinhardtii* (XM_001695212.1), *G. max* (XM_003533929.1), *Micromonas* (XM_002499681.1), *O. lucimarinus* (XM_001419901.1), *P. patens* (XM_001777362.1), *P. trichocarpa* (XM_002305227.1), *S. moellendorffii* (XM_002981360.1), *V. carteri* (XM_002947069.1).

Analysis of the nucleotide sequence of the cloned genomic DNA fragment (Accession No. JQ735969) indicated that *dshsp90* was 8.79 kb (Figure S1A), which might be the longest genomic sequence of Hsp90s (Figure 3). A comparison of the genomic sequence with the cDNA sequence of *dshsp90* revealed that the ORF of *dshsp90* contained 21 exons interrupted by 20 introns with an average size of 313 bp, which is longer than the mean length of higher plant introns (249 bp) [24] but smaller than that of *C. reinhardtii* (373 bp). The smallest intron was 108 bp (intron 19), while the largest one was 707 bp (intron 15). The 21 exons spread over the 8.79 kb region and ranged in the size from 35 bp to 241 bp with an average size of 110 bp (Table 1), which is much smaller than the mean length of 183 bp in higher plants [24]. Six of the splice junctions were type I, nine were type II, and five were type 0. There were 7 splice types found in *dshsp90*, and only 2 splice junctions followed the typical canonical consensus GT-AG dinucleotide sequence (Table 1). The boundary structures of the 18 other introns were distributed among 8 TG-GG type, 3 TA-GG type, 2 GC-GT type, 2 TC-GG type, 1 GT-GA type, 1 AA-GT type and 1 GC-GG type. The types of splice site of *dshsp90* were much more complicated than the other algae *hsp90s*. For example, the 7 introns of *hsp90* from *C. reinhardtii* contain 3 TG-GG type, 2 GT-AG type, 1 GT-GG type and 1 GT-GC type, while the 9 introns of *hsp90* from *V. carteri* have 3 TG-GG, 2 GT-AG, 2 TA-GG, 1 AG-GT and 1 AG-TA borders. Alternate splicing of exons can occur between splice junctions of the same type. To examine whether alternate splicing occurred in *dshsp90*, Northern blot analysis was performed and only a single band could be obtained, implying there was a single specie of mRNA (Figure S2). Moreover, Western blot analysis also indicated that only one form of protein was expressed in the *D. salina* cells under various conditions (data not shown).

Alignment of the exon sequence of the *dshsp90* gene with the other plant *hsp90* genes showed that the *dshsp90* gene has the most complicated gene structure. Although *D. salina* and *C. reinhardtii* have a close evolutionary relationship and DsHsp90 and CrHsp90A shared a high sequence homology of 83.3%, the gene structures between these two algae were quite different (Figure 3). The gene of *hsp90* from *C. reinhardtii* has much fewer introns (only 7). The other plant *hsp90* genes also contained less than 10 introns. For example, there are 9 introns for *hsp90* from *Volvox carteri f. nagariensis*, 4 for *Coprinopsis cinerea okayama* and *Schizophyllum commune*, 3 for *Micromonas* and *Selaginella moellendorffii*, 2 for *Oryza sativa*, *Arabidopsis thaliana* and *Ostreococcus lucimarinus*, and only 1 for *Physcomitrella patenshave*. Although the debate regarding the origin of introns in eukaryotic cells has lasted for a long time between the introns-early and introns-late concepts, a hypothesis combining both concepts has been proposed to explain the mechanism of intron evolution in eukaryotes, which involves intron gain, intron loss and intron slide [25–27]. Moreover, the genomes of single-cell eukaryotes are usually intron-poor when compared to the intron-rich multicellular organisms [28]. However, the genomes of algae seem to be intron-rich although the mean intron length of the multicellular *V. carteri* is longer than the single-cellular *C. reinhardtii* [29,30]. Compared with *hsp90s* from *C. reinhardtii* and *V. carteri*, the *hsp90* gene from *D. salina* was unusually complicated, and all of the three well-structured domains of DsHsp90 were interrupted by several introns. It is unclear yet how the intron number and size differ in the genomes of *D. salina* and *C. reinhardtii*, and whether the large number of introns also exists in the other genes from *D. salina*. Nonetheless, the extremely large number of the introns of *dshsp90* suggested that it might be a highly expressed gene containing more introns with regulatory roles since the highly expressed genes in plants are usually less compact and contain more and long introns compared to the low expression genes [31].

**Figure 4:**
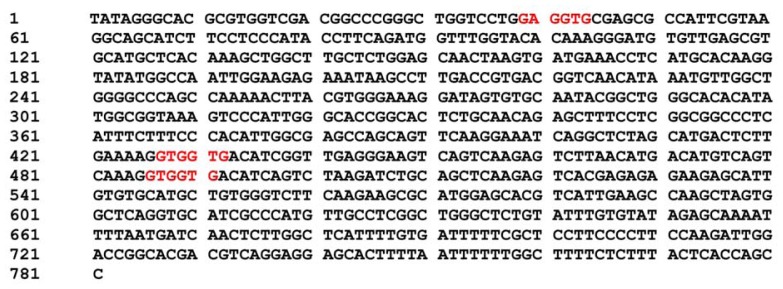
Sequence of the promoter region of *dshsp90*. The three Alfin1 putative binding sites with the motif of GTGGNG or GNGGTG are highlighted in red.

To identify the potential regulatory factors involved in *dshsp90* gene expression, sequence analysis was performed of the 5′ upstream region of the *dshsp90* gene. The regulatory elements were analyzed by the online software plantCARE [32], and the results are shown in Figure S3. The promoter region of *dshsp90* beginning at −781 bp contained several canonical *cis*-acting elements such as CAAT box and TATA box to ensure the transcription start. Putative *cis*-elements responding to light, gibberellin, MeJA, abscisic acid (ABA) and cold stimulation were also identified. The existence of response elements to ABA and MYB binding site and the lack of the other stress-responsive elements such as DRE/CRT suggested that the expression of *dshsp90* might be induced by stresses via the ABA-dependent pathway [33]. The putative transcription factor (TF) binding site was also analyzed by TFSEARCHER [34], which indicated that the promoter of *dshsp90* had potential heat shock transcription factor (HSF) binding sites. It is worth noting that three Alfin1 putative binding sites were identified with the motif of GTGGNG or GNGGTG (Figure 4). Alfin1 has been shown to be a novel TF that binds to the promoter of salt-inducible genes [35]. Thus the *in silico* analysis suggested that *dshsp90* might be involved in the salt response of the *D. salina* cells.

### 2.2. Biophysical and Biochemical Characterization of Recombinant DsHsp90

**Figure 5:**
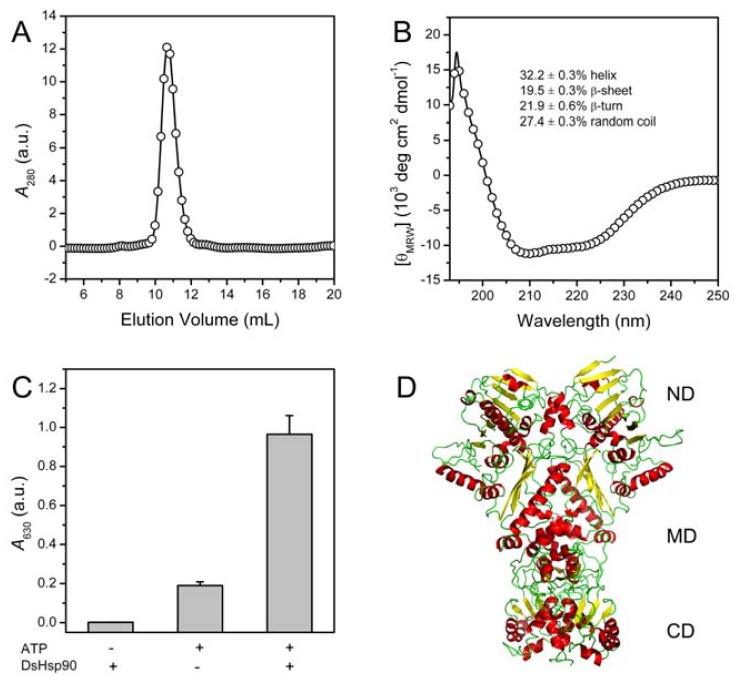
Biophysical and biochemical characterization of DsHsp90. (**A**) SEC profile of the recombinant DsHsp90 using a superdex G200 10/30 column. The apparent molecular weight was calculated from the standard curve obtained from the standard markers provided by GE Healthcare; (**B**) Far-UV CD spectrum of DsHsp90. The percentages of the secondary structures were determined using the CONTINLL, SELCON3 and CDSSTR algorithms within the CDPro analytical software [37]. The results are presented as the average ± standard error calculated from the values of the three methods; (**C**) ATPase activity of DsHsp90. The enzyme assay was conducted in the presence or absence of 5 mM ATP or 20 μg DsHsp90; (**D**) Predicted three-dimensional structure of DsHsp90 by SWISS-MODEL using the crystal structure of yeast Hsp90 (PDB ID: 2CG9) as the template structure. ND, MD and CD are the *N*-terminal domain, middle domain and *C*-terminal domain, respectively.

The analysis of the amino acid sequence indicated that DsHsp90 belongs to the Hsp90A family. To confirm this proposal, the recombinant DsHsp90 was produced in *E. coli*, and was purified to a homogenous state with only one single peak in the SEC elution profile (Figure 5A). From the SEC elution volume, the apparent molecular weight (MW) of DsHsp90 was calculated to be 191 kDa, which was slightly larger than the actual MW value of the dimeric DsHsp90 (159 kDa). This is consistent with the fact that the shape of the Hsp90A molecules deviated from the sphere shape [36], which will affect its position in the SEC profile. Nonetheless, it is clear that DsHsp90 mainly existed as a dimer in solution.

The structural features of DsHsp90 were evaluated by CD spectroscopy, which revealed that the recombinant DsHsp90 was well-folded containing both α-helical and β-sheet structures (Figure 5B). The percentages of the secondary structures were calculated by CDpro [37], and the three algorithms used for prediction gave rather consistent results. The predicted values are quite consistent with the crystal structures of Hsp90As. For example, the structure of yeast Hsp90 is composed of 32.3% α-helix and 310-helix, 16.7% β-sheet and 50.9% other secondary structures [36]. Since Hsp90s are ATPases, we also examined whether recombinant DsHsp90 possessed ATPase activity using the malachite green reagent method [38]. The data shown in Figure 5C clearly indicated that DsHsp90 could exhibit ATPase activity as revealed by the significant increase in the absorbance at 630 nm when compared to the controls that either lacked the substrate ATP or the enzyme DsHsp90. The three-dimensional structure of DsHsp90 was modeled using SWISS-MODEL using the yeast Hsp90 crystal structure [36] as the template. The modeled structure of DsHsp90 (Figure 5D) shared similar structural features to the Hsp90A family.

### 2.3. Relative Expression of the Dshsp90 Gene

**Figure 6:**
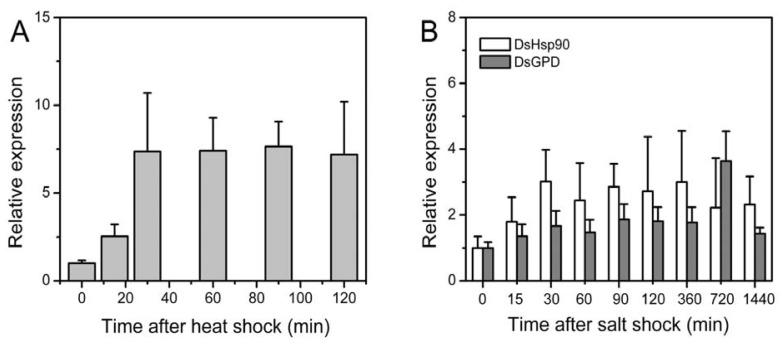
Relative fold increase of the expression level of *dshsp90* and *dsgpd* transcripts during stress evaluated by quantitative RT-PCR. (**A**) Time-course study of the transcript level of *dshsp90* after heat shock. Real-time quantitative PCR was carried out using the total RNA extracted from the *D. salina* cells after heat treatment at 37 °C for 0–120 min; (**B**) Time-course study of the transcript levels of *dshsp90* and *dsgpd* after salt shock from 2 M to 4 M NaCl for 0–24 h. The expression level of *ds18S* was used as an internal control.

The *in silico* analysis above indicated that *dshsp90* might be a heat- or salt-inducible gene. To verify this proposal, real-time quantitative PCR was used to analyze the relative expressions of *dshsp90* under heat and salt stress. As shown in Figure 6A, the transcriptional level of DsHsp90 increased immediately after heat shock, and reached its maximum after 30 min heat shock treatment. The maximum level of the *dshsp90* transcript was about 7-fold of that without heat treatment. This observation confirmed that *dshsp90* responded early upon heat shock, which might facilitate to stabilize its client proteins.

The *D. salina* cells have been shown to grow optimally in ~2 M NaCl [39], and a salt shock was performed by transferring the living conditions of the *D. salina* cells from 2 M to 4 M NaCl. Similar to the heat shock results, the amount of the *dshsp90* transcripts increased immediately upon the salt stress and reached maximum after 30 min treatment (Figure 6B). The expression of *dshsp90* was maintained at a high level for the duration of our experimental time of 24 h. Meanwhile, the expression level of *dsgpd* was not altered significantly during the first 6 h cultivation in 4 M NaCl, increased about three-fold at 12 h, and dropped to the normal level at 24 h. One possible explanation of the minor increase of the expression level of *dsgpd* during the first 6 h is that the osmotic-stress induced gene expression usually occurs 12–24 h after the salt shock [2], while the other possible reason is that the *Dunaliella* cells have already accumulated considerable amounts of glycerol to fight against the osmotic pressure under 2 M NaCl [6]. Nonetheless, the observations in Figure 6 suggested that *dshsp90* was an early response gene to heat or salt stress. The high *dshsp90* expression level under stress conditions also implied that DsHsp90 was essential for the *Dunaliella* cells to survive under extreme conditions.

## 3. Experimental Section

### 3.1. Chemicals

Tris, sodium dodecylsulfate (SDS), isopropyl-1-thio-β-d-galactopyranoside (IPTG), ATP, ammonium molybdate, malachite green oxalate, hydrochloride acid and tween-20 were Sigma products. All other chemicals were local products of analytical grade or higher. All restriction enzymes used in this study were purchased from TaKaRa (Shiga, Japan). The SMART™ RACE cDNA Amplification Kit was from Clontech (Palo Alto, CA, USA). The PMD18-T vector, LA Tag and RNAiso Plus kit was from TaKaRa (Shiga, Japan).

### 3.2. Algae and Growth Conditions

The green algae *D. salina* strain was bought from the Institute of Hydrobiology, the Chinese Academy of Sciences. The *D. Salina* cells were grown at 25 °C, illuminated at 4500 lux with a long-day condition (18 h light and 6 h dark) in medium comprising 2 M NaCl, 50 mM NaHCO_3_, 2.5 mM NaNO_3_, 5 mM MgSO_4_, 0.2 mM KH_2_ PO_4_, 6 μM EDTA, 2 μM FeCl_3_, 0.2 mM CaCl_2_, 7 μM MnCl_2_, 1 μM ZnSO_4_, 1 μM Co(NO_3_)_2_ and 1 μM CuSO_4_. The pH was adjusted to 7.5 using 2 M HCl. The *Dualiella salina* cells used in this study were collected at their logarithmic phase.

### 3.3. Gene Cloning and Sequence Analysis

The genome of *D. salina* was extracted by cetyltrimethylammonium bromide (CTAB) using the standard protocol [40], and the RNA was isolated by RNAiso Plus. The full length cDNA was obtained using the SMARTer™ RACE cDNA Amplification Kit. The whole cDNA was divided into two segments with an overlap, and two pairs of primers were designed accordingly to amplify these two segments by step-down PCR method (Table S1). PCR was run using the following conditions: 1cycle of denaturation at 95 °C/4 min followed by 30 three-round cycles of amplification (95 °C/30 sec, 59 °C, 56 °C, 53 °C/30 sec (5/5/20 cycles), 72 °C/3 min), finally extension at 72 °C/10 min. The PCR products were constructed to the pMD19-T vector, and were confirmed by DNA sequencing (Invitrogen, Shanghai, China). The boundaries of the introns and exons were identified by the Vector NTI 11 software. The promoter sequences were cloned by adaptor PCR using the genome walking method [41,42], and the GSP primers were designed according to the 5′-UTR region. The sequence was analyzed using plantCARE [32,43].

### 3.4. Material Treatments and Total RNA Isolation

50 mL algae cells in the logarithmic phase were collected for further processing. The heat shock treatment was performed by transferring the cells from 25 °C to 37 °C with the temperature controlled by water bath. The samples used for further time-course analysis were gathered after 15 min, 30 min, 60 min, 90 min and 120 min heat shock treatment. The salt shock was carried out by adding 5.844 g NaCl to 50 mL algae cell solutions, and the final concentration of NaCl was 4 M. The collected cells were treated by liquid nitrogen before total RNA isolation. RNA was isolated by RNAiso Plus, and contaminating DNA was degraded by treating each sample with RNase-free DNase. The total RNA was quantified by optical density and the quality was evaluated by gel electrophoresis by the intactness of the 28S and 18S rRNA subunit. One μg total RNA was reverse transcribed using ReverTra Ace (Toyobo, Japan) and random primer according to the manufacturers’ recommendations. The obtained cDNA was diluted 1:20 with H_2_O prior to the quantitative PCR analysis.

### 3.5. Quantitative PCR with SYBR Green

The relative gene expressions of DsHsp90 and DsGPD (Accession No.AY845323.1) in *D. salina* were analyzed by real-time quantitative reverse-transcription PCR. The relative abundance of the *ds18S* gene (Accession No. EF195157.1) was also determined and used as the internal control. For each transcript, a standard curve was constructed using the purified PCR product generated for each specific primer pair. The 20 μL reaction solutions contained 4 μL diluted cDNA, 10 pmol primer, 4 μL H_2_O and 10 μL SYBR Green real time PCR master mix (TOYOBO, Japan). The real time qPCR was run on iQ5(Bio-Rad) with 1 cycle of denaturation at 95 °C/10 min, followed by 40 two-segment cycles of amplification (95 °C/30 sec, 55 °C (18S and GPD) or 57 °C (Hsp90)/15 sec, 72 °C/15 sec). The fluorescence was measured during PCR and one three-segment cycle of product melting (95 °C/1 min, 55 °C/30 sec, 95 °C/30 sec). The baseline adjustment method of the Bio-RadiQ5 software was used to determine the Ct in each reaction. A melting curve was constructed for each primer pair to verify the presence of one gene-specific peak and the absence of primer dimer. All samples were repeated three times.

### 3.6. Protein Expression and Purification

After digestion of the PCR product and of the plasmid pET28a with NdeI and BamHI, the *dshsp90* gene was ligated into the expression vector pET28a (Novagen). The six-His Tag sequence of pET28a vector was fused to the N-terminus of the open reading frame for further purification. The recombinant plasmids were then transformed into *E. coli* BL21(DE3) (Novagen). The recombinant strain was inoculated into 5 mL of Luria–Bertani medium supplemented with 50 μg/mL kanamycin and grown overnight at 200 r/min and 37 °C. The cultures were diluted (1:100) in the same medium and grown at 37 °C to reach an OD value of 0.6–0.8, and then the recombinant protein expression was induced by the addition of 1 mM IPTG. After 4 h induction at 37 °C, the cultures were harvested and sonicated. The His-tagged recombinant proteins in the soluble fractions were first purified by the metal-chelated affinity chromatography using a 1 mL Pharmacia Ni-NTA His-Bind column, followed by a further step of purification by Mono Q 5/50 GL anion-exchange chromatography. The final products were collected after purification by size exclusion chromatography (SEC) using a Superdex G200 column equipped on an ÄKTA purification system. The buffer used for gel filtration contained 40 mM HEPES and 25 mM KCl, pH 7.5. The purified proteins were found to be homogeneous by SDS-polyacrylamide gel electrophoresis (SDS-PAGE) and SEC analysis, and the purity of the final products was above 98%.

### 3.7. ATPase Activity Assay

The ATPase activity of DsHsp90 was determined by the liberation of inorganic phosphate using a modified procedure [44] of the malachite green reagent method [38]. In brief, the working reagent to terminate the reaction was freshly prepared by mixing 130 mM Malachite Green, 7.5% (w/v) ammonium molybdate and 11% (v/v) Tween-20 with a volume ratio of 10:2.5:0.2. The reaction solution contained the enzyme in 40 mM HEPES and 100 mM KCl, pH 7.5, with the addition of 5 mM ATP. The reaction was terminated after 30 min reaction by adding 400 μL hydrochloride acid, 500 μL water and 250 μL working reagent in 100 μL reaction solutions. After 10 min equilibrium, the absorbance at 630 nm was recorded.

### 3.8. Spectroscopy

The samples used for spectroscopic experiments were prepared by dissolving the protein in 50 mM Tris-HCl buffer containing 25 mM KCl, pH 7.5, with a final protein concentration of 0.2 mg/mL. Far-UV circular dichroism (CD) spectra were recorded on a Jasco 715 spectrophotometer with a 1 mm pathlength cell. The resultant spectrum was baseline-subtracted *versus* the buffer spectrum. The percentages of the secondary structures were determined from the CD spectrum with a wavelength range of 200–250 nm using the CONTINLL, SELCON3 and CDSSTR algorithms within the CDPro analytical software [37]. The results were presented as the average ± standard error calculated from the values of the three methods.

### 3.9. Homology Modeling

The automated protein structure homology modeling was performed using the online software SWISS-MODEL in the project mode [45]. The crystal structure of Hsp90 from *Saccharomyces cerevisiae* (PDB ID: 2CG9) [36] was used as the template structure. The model structure of DsHsp90 was manipulated and rendered in PyMol (Available online: http://www.pymol.org/).

## 4. Conclusions

In this research, we cloned the *dshsp90* gene encoding a polypeptide composed of 696 amino acids from the halotolerant unicellular green algae *D. salina*. Sequence alignment indicated that DsHsp90 shares high homology to the cytosolic Hsp90 family with conserved motifs characteristic of Hsp90A. Further biophysical and biochemical studies of the recombinant protein revealed that DsHsp90 possessed ATPase activity and existed as a dimer with similar percentages of secondary structures to those well-studied Hsp90As. Thus the gene was named *dshsp90* according to its structure and function relationship to the Hsp90A family. Analysis of the nucleotide sequence of the cloned genomic DNA fragment indicated that *dshsp90* contained 21 exons interrupted by 20 introns, which is much more complicated than the other plant *hsp90* genes. Such a large number of introns of *dshsp90* suggested that it might be a highly expressed gene containing more introns with regulatory roles. The promoter region of *dshsp90* contained putative *cis*-acting stress-responsive elements and binding sites of transcriptional factors that respond to heat shock and salt stress. Further experimental study confirmed that the *dshsp90* transcripts were induced early by heat shock and salt shock, suggesting that *dshsp90* was involved in the early response of the *D. salina* cells against environmental stresses. The characterization of *dshsp90* provides a starting point for further investigation of the role of *dshsp90* in the haloadaption mechanism of *D. salina*.

## Supplementary Materials



## Figures and Tables

**Table 1:** Nucleotide sequence at the splice junction sequences and sizes of exons and introns of *dshsp90*.

Exon No	Exon Size(bp)	Intron Size(bp)	Boundary Sequence				Junction Type

			Exon	5′-Intron	3′-Intron	Exon	
1	98	199	TCTCTG	TGAGTC	TGCAGG	ATCATC	0
2	43	115	TGCGGG	TGAGTG	CGCAGG	ATGTGA	I
3	42	206	TCAGGT	GCGCGC	GCAGGT	TCATGA	I
4	56	330	ATCAGG	TCCGTC	TGCAGG	ATCATC	0
5	64	221	CCAAGG	TCAGTA	TTCAGG	CTGACC	I
6	35	511	GCCAGG	TGAGGG	TGCAGG	AGTGGA	0
7	241	307	TGAAGG	TGTGTG	TGCAGG	AGGACC	II
8	144	275	AGAAGG	GTGGGT	AGTCAG	AGGATG	II
9	113	464	ATCTGG	TAAGCC	TCCAGG	ATGCGT	I
10	124	448	TCAAGT	GCGTGT	GCAGGT	CCATCC	II
11	144	163	TCAAGG	TGAGGT	CTGAGG	GTATTG	II
12	75	289	AGTTGA	GTAGAA	AGGTGA	TCAAGA	II
13	108	179	AGCTGG	TAGGTA	CGCAGG	GTGTGC	II
14	150	263	ACTGGT	AAGGAG	TCAGGT	GAGAGC	I
15	125	707	TGATGG	TGAGTG	TGCAGG	CAAGAA	I
16	137	580	AGGTGG	GCGTCC	AGGTGG	AGAAGG	II
17	181	277	TCATGG	TGGGTG	TGCAGG	GTGAGC	II
18	51	243	TCAAGG	TAAGGG	TGCAGG	ATCTAG	II
19	98	108	ATCAAG	GTAGGT	CTACAG	CTTGGC	0
20	97	373	TGGAGG	TGAGTC	TGCAGG	ATGTCG	0
21	180						
