# Supplementary material for: DsHsp90 Is Involved in the Early Response of Dunaliella salina to Environmental Stress†
Source: Int J Mol Sci. 2012 Jun 27;13(7):7963–79. doi: 10.3390/ijms13077963 (PMC3430215; doi:10.3390/ijms13077963)

DsHsp90 Is Involved in the Early Response of *Dunaliella salina* to Environmental Stress

Supplementary Materials

Table S1. Primers used for gene cloning and promoter analysis.

**Primers**	**Primer Sequence**
First Primer For	5'-TCACCAGCCACAATGAGCAACACAG-3'
First Primer Rev	5'-TTGCCAAATGCCTCATAGAACT-3'
Second Primer For	5'-TGTATGTGCGCCGTGTGTTC-3'
Second Primer Rev	5'-TCCACCCTCCCAAATAAAATG-3'
Long Adaptor	5'-GTAATACGACTCACTATAGGGCACGCGTGGTCGACGGCCC GGGCTGGT-3'
Short Adaptor	5'-($PO_{4)}$ACCAGCCCA(NH_4)-3'
AP1	5'-GTAATACGACTCACTATAGGGC-3'
AP2	5'-ACTATAGGGCACGCGTGGT-3'
GSP1	5'-AACTCCCGCAGCCAGATTTC-3'
GSP2	5'-GGCTGGTGAGTAAAGAGAAAAG-3'
18S For	5'-GCCTGAGAAACGGCTACCACA-3'
18S Rev	5'-CTACGAGCTTTTTAACTGCAACAAC-3'
DsHsp90 For	5'-CTCCGATGCTCTGGATAAAATCA-3'
DsHsp90 Rev	5'-TCGGGGTGCTTGGTGGTCACAG-3'
DsGPD For	5'-CTGAAGGCTCGTGGAGTGGA-3'
DsGPD Rev	5'-TGCCAGGACATGGTGTTGC-3'

Figure S1. Characterization of the gene structure of *dshsp90*. (**A**) DNA gel electrophoresis of the two genomic segment of *dshsp90* using the primers listed in Supplemental Table 1. The marker is 15000 bp, 10000 bp, 7500 bp, 5000 bp, 3000 bp, 1500 bp and 1000 bp, from the top to the bottom, respectively. Lane 1, the PCR product from first pair of primers. Lane 2, the single primer control of first For primer. Lane 3, the single primer control of first Rev primer. Lane 4, the PCR product from second pair of primers. Lane 5, the single primer control of second For primer. Lane 6, the single primer control of second Rev primer; (**B**) DNA gel electrophoresis of the promoter of *dshsp90*. The marker is 2000 bp, 1000 bp, 750 bp, 500 bp, 250 bp and 100 bp, from the top to the bottom, respectively. Lane 1, nest PCR product of the promoter, the specific line is about 780 bp. Lane 2, the single primer control of AP2. Lane 3, the single primer control of GSP2.

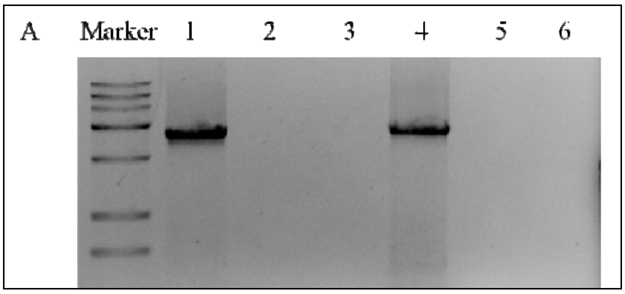

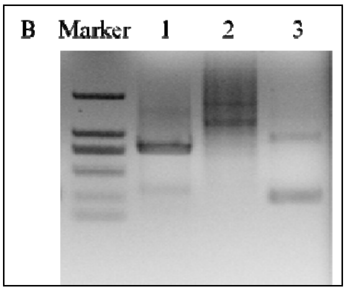

Figure S2. Northern Blot analysis revealed that there was only one specie of mRNA in the *D. salina* cells. 30 μg total RNA was loaded per lane. Lane 1, *D. salina* cells cultured in 2 M Nacl at 23 °C. Lane 2, *D. salina* cells cultured in 2 M NaCl at 23 °C was transferred to 37 °C, and the total RNA was extract after 30 min cultivation. Lane 3, *D. salina* cells cultured in 2 M NaCl was transferred to 4 M NaCl, and the total RNA was extract after another 30 min cultivation. The digoxigenin (DIG)-labeled probes were generated using the DIG DNA labeling and Detection Kit (Roche), and the probe sequence is CTCTGTCCGCTGGTGCTGATGTGTCCATGATTGGGCAGTTCGGTGTGGGCTTC TACTCTGCCTACCTTGTGGCTGACCGCGTGACTGTGACCACCAAGCACCCCGAT GATGAGCAGTACACCTGGGAGTCCCAGGCTGGTGGCTCCTTCACCGTCACCCGT GACACTGAAGGCGAGCAGCTGGGCCGTGGCACCAAGATTGTGCT.

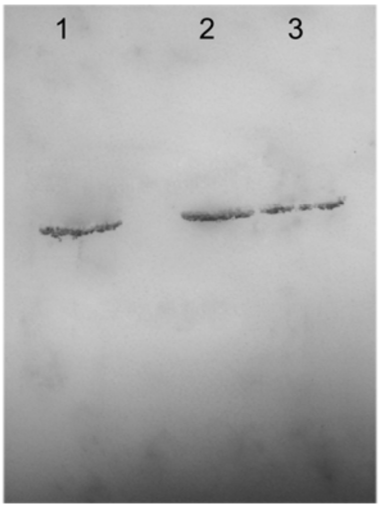

Figure S3. Analysis of the *cis*-acting elements in the promoter region of *dshsp90* by by the online software plantCARE (http://bioinformatics.psb.ugent.be/webtools/plantcare/html/).

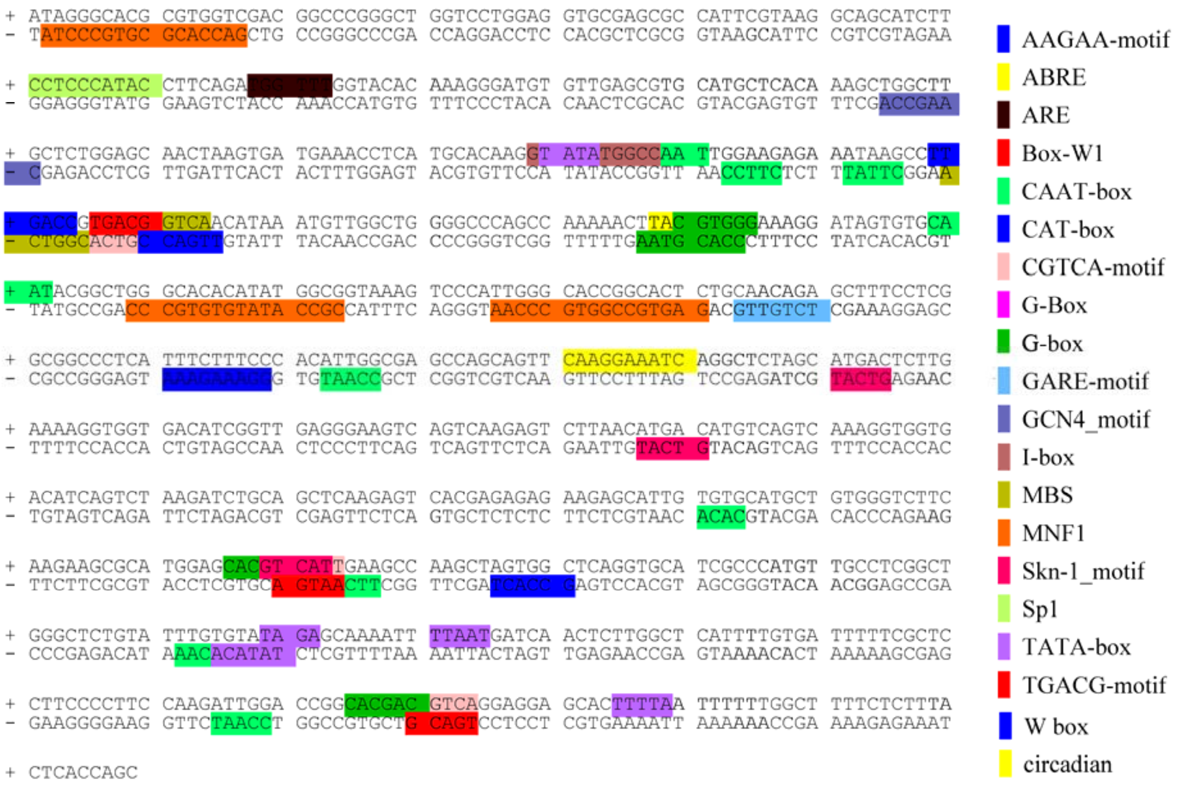

Supplement: Supplementary file 1 [file ijms-13-07963-s002.pdf]
